# SPT5 regulates RNA polymerase II stability via Cullin 3–ARMC5 recognition

**DOI:** 10.1126/sciadv.adt5885

**Published:** 2025-01-24

**Authors:** Yuki Aoi, Leila Iravani, Isabella C. Mroczek, Sarah Gold, Benjamin C. Howard, Ali Shilatifard

**Affiliations:** Simpson Querrey Institute for Epigenetics, Department of Biochemistry and Molecular Genetics Feinberg School of Medicine, Northwestern University, Chicago, IL 60611, USA.

## Abstract

The stability of RNA polymerase II (Pol II) is tightly regulated during transcriptional elongation for proper control of gene expression. Our recent studies revealed that promoter-proximal Pol II is destabilized via the ubiquitin E3 ligase cullin 3 (CUL3) upon loss of transcription elongation factor SPT5. Here, we investigate how CUL3 recognizes chromatin-bound Pol II as a substrate. Using an unbiased proteomic screening approach, we identify armadillo repeat-containing 5 (ARMC5) as a CUL3 adaptor required for VCP/p97-dependent degradation of SPT5-depleted, chromatin-bound Pol II. Genome-wide analyses indicate that ARMC5 targets promoter-proximal Pol II in a BTB domain–dependent manner. Further biochemical analysis demonstrates that interaction between ARMC5 and Pol II requires the transcriptional cyclin-dependent kinase 9 (CDK9), supporting a phospho-dependent degradation model. We propose that defective, promoter-proximal Pol II that lacks SPT5 is rapidly eliminated from chromatin in a noncanonical early termination pathway that requires CDK9-dependent interaction with the CUL3-ARMC5 ubiquitin ligase complex.

## Teaser

Phospho-dependent degradation mediated by cullin 3 with ARMC5 adaptor eliminates defective transcription complex lacking SPT5.

## INTRODUCTION

The process of transcriptional elongation by RNA polymerase II (Pol II) is critical for all eukaryotic gene expression, and its regulation plays a crucial role in disease and development (*1*). In metazoans, Pol II is subject to promoter-proximal regulation governed by several evolutionally conserved transcription elongation factors (*1*, *2*). Following promoter escape, promoter-proximal Pol II is actively engaged but pauses at the +1 nucleosome, where it associates with the negative elongation factor (NELF) complex and the SPT4-SPT5 heterodimer often referred to as DRB sensitivity-inducing factor (DSIF) (*3*–*6*). Positive transcription elongation factor b (P-TEFb), which consists of cyclin-dependent kinase 9 (CDK9) and cyclin T1, is essential for subsequent release of promoter-proximal paused Pol II into productive elongation (*7*). Upon CDK9-induced pause-release activation, Pol II–associated SPT6 functions to recruit the PAF1 complex, which renders the elongating Pol II complex capable of processive transcription throughout the long distances of gene bodies (*8*–*10*).

SPT5 physically interacts with the core Pol II complex throughout the elongation stage and plays various roles in transcription (*1*, *11*). SPT5 regulates promoter-proximal pausing via recruitment of NELF and promotes the rate of elongation downstream of the promoter-proximal release (*4*, *12*–*14*). These pleiotropic functions are regulated by phosphorylation at distinct SPT5 domains (*15*, *16*). Recently, we and others have also reported a key role for SPT5 in stabilization of promoter-proximal Pol II (*14*, *15*). These features of SPT5 stand in contrast to those of NELF, which only occupies chromatin in association with promoter-proximal Pol II at most genes, functions to regulate transcription elongation, and is required for cell growth and development (*6*, *17*–*19*).

Regulation of Pol II stability has been extensively studied in the context of DNA damage response. Upon ultraviolet irradiation, Pol II stalled at DNA lesions is eliminated through ubiquitin-mediated degradation as a last resort for transcription recovery (*20*). This degradation process includes ubiquitination of the Pol II subunit RPB1 catalyzed by the E3 ubiquitin ligase cullin 4 (CUL4), which is associated with several proteins including the core subunit DDB1 and the Cockayne syndrome proteins CSA and CSB (*20*). CUL4 uses CSA as an adaptor to recognize Pol II and has access to multiple RPB1 lysine residues, including K1268, for ubiquitylation (*21*–*23*). In contrast, we have recently shown that RPB1 degradation upon SPT5 loss is mediated by the distinct ubiquitin E3 ligase cullin 3 (CUL3) (*14*). In the absence of SPT5, Pol II undergoes VCP/p97-dependent dissociation from chromatin following recruitment of CUL3 to promoter-proximal regions (*14*).

How CUL3 targets promoter-proximal Pol II is currently unknown. CUL3 is known to recognize specific substrates via adaptor proteins called BTB proteins, which interact with CUL3 via their BTB (Broad-Complex, Tramtrack, and Bric a brac) domains (*24*). The human genome contains nearly 200 genes encoding BTB proteins (*25*), suggesting that a BTB protein likely serves as an adaptor that enables CUL3 to recognize promoter-proximal Pol II as a substrate. The BTB protein armadillo repeat-containing 5 (ARMC5) was recently shown to associate with RPB1 and function to regulate the bulk Pol II levels via its participation in an RBX1-CUL3 E3 ubiquitin ligase complex (*26*, *27*), but its role in transcriptional elongation control is not clear. ARMC5 variants are found in patients with primary macronodular adrenal hyperplasia, a rare cause of Cushing’s syndrome (*28*).

How CDK9 regulates degradation of promoter-proximal Pol II is also largely unknown. We have recently shown that CUL3-dependent Pol II degradation requires the kinase activity of CDK9 (*14*) but not its typical transcription activator partners within BRD4-PTEFb or SEC (*14*). Here, we performed an unbiased proteomic screen in search of a CUL3 adaptor that enables Pol II recognition in response to SPT5 loss. We identify ARMC5 as a CUL3 adaptor required for CUL3 targeting of promoter-proximal Pol II and find that ARMC5–Pol II interaction is regulated by CDK9.

## RESULTS

### The CUL3 adaptor ARMC5 mediates degradation of Pol II upon SPT5 loss

**Fig. 1.**
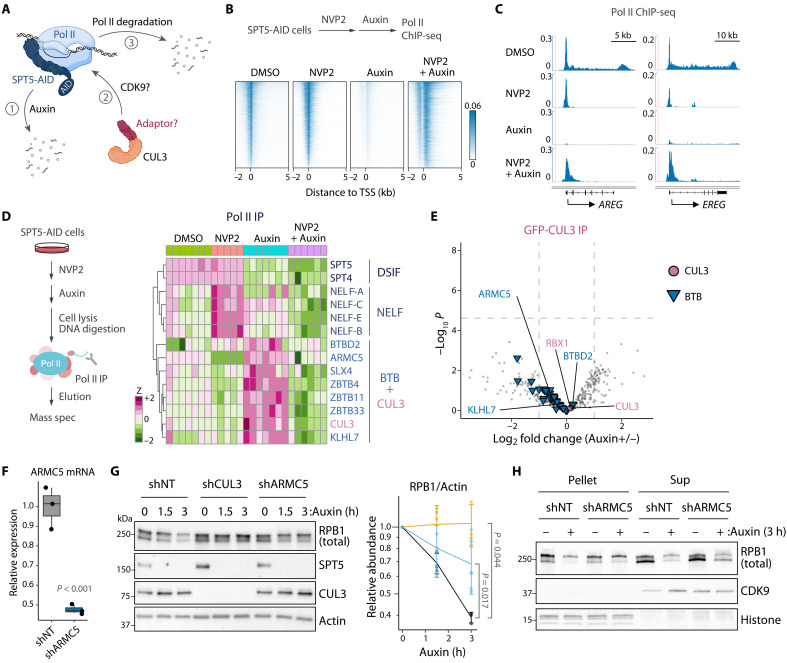
The CUL3 adaptor ARMC5 mediates Pol II degradation upon SPT5 loss. (**A**) Schematic of CUL3-mediated Pol II degradation upon SPT5 loss. (**B** and **C**) TSS-centered signal heatmaps (B) and representative signal tracks (C) for Pol II ChIP-seq in SPT5-AID cells treated with NVP2 (250 nM, 2 hours) followed by auxin (500 μM, 3 hours). *N* = 6481. (**D**) Clustering of select proteins quantified in Pol II IP-MS analysis. Protein abundance is shown as *z* score. *N* ≥ 5. (**E**) Volcano plot showing GFP-CUL3 IP-MS analysis in SPT5-AID cells treated with auxin (500 μM, 3 hours). Dotted lines indicate twofold change and adjusted *P* = 0.01. *N* = 5, limma test. (**F**) ARMC5 mRNA levels quantified in RNA-seq DESeq2 analysis. *N* = 3, Wald test. (**G**) Western blot analysis of whole-cell extracts from SPT5-AID cells treated with CUL3 or ARMC5 shRNA followed by time-course auxin treatment (500 μM). Quantification of RPB1 protein is also shown (shNT, black; shCUL3, orange; shARMC5, blue). *N* ≥ 3, Tukey’s test. Error bars, SD. (**H**) Western blot analysis of pellet and supernatant fractions of nuclei isolated from SPT5-AID cells treated with ARMC5 shRNA followed by auxin treatment (500 μM, 3 hours). Histone (ponceau stain) and CDK9 serve as chromatin and nuclear markers, respectively. Figure 1A was created with BioRender.com (agreement #BD27KXXWOH).

To search for an adaptor protein that enables CUL3 to recognize promoter-proximal Pol II upon SPT5 loss (Fig. 1A), we sought to perform an unbiased proteomic screen using Pol II coimmunoprecipitants from SPT5-depleted cells. However, we have previously shown via chromatin immunoprecipitation–sequencing (ChIP-seq) that rapid depletion of auxin-inducible degron (AID)–tagged SPT5 proteins by auxin treatment leads to a genome-wide reduction of Pol II chromatin occupancy in human DLD-1 cells (Fig. 1, B and C). Therefore, to improve the efficiency of identification, we chose to leverage the fact that degradation of SPT5-depleted Pol II relies on the kinase activity of CDK9 (*14*). Pol II chromatin occupancy is largely restored when auxin treatment is preceded by pretreatment with the CDK9 inhibitor NVP-2 (Fig. 1, B and C). Given this requirement for CDK9 activity, we reasoned that NVP-2 pretreatment would abolish any interaction between Pol II and a CUL3 adaptor that would otherwise be induced by auxin treatment in SPT5-AID cells (Fig. 1A).

For the unbiased screen, we performed quantitative Pol II immunoprecipitation–mass spectrometry (IP-MS) experiments using SPT5-AID cells treated with NVP-2 and/or auxin (Fig. 1D and table S1). To validate the screen, we first examined the relative abundance of transcription elongation factors associated with Pol II. As expected, SPT5 and its DSIF heterodimer partner SPT4 were largely lost upon auxin treatment (Fig. 1D). All four NELF subunits (NELF-A, NELF-B, NELF-C, and NELF-E) were enriched as expected upon treatment with NVP-2 alone, whereas additional auxin treatment led to NELF loss (Fig. 1D). Western blots for SPT5 and NELF-C confirmed the differential interactions observed in MS data (fig. S1A). These results are in agreement with the previous findings that CDK9 inhibition blocks pause release and that NELF recruitment to Pol II depends on SPT5 (*1*).

Next, we examined the relative abundance of CUL3 and BTB proteins (BTBs) detected among Pol II coimmunoprecipitants. Auxin treatment resulted in enrichment of CUL3 and seven BTBs that were lost when auxin treatment was preceded by pretreatment with NVP-2 (Fig. 1D). To determine which of these seven Pol II–interacting BTBs also interact with CUL3, we performed green fluorescent protein (GFP) IP-MS in SPT5-AID cells expressing GFP-tagged CUL3. In addition to RING box protein 1 (RBX1), a core component of the CUL3-RBX1 E3 ubiquitin ligase complex, more than 30 BTBs were identified among CUL3 coimmunoprecipitants, including three Pol II–associated BTBs: BTBD2, ARMC5, and KLHL7 (Fig. 1E; fig. S1, B and C; and table S1). SPT5 depletion by auxin did not significantly alter CUL3 interaction with RBX1 or any of these BTBs (Fig. 1E).

Following identification of BTBD2, ARMC5, and KLHL7 as potential CUL3 adaptors involved in Pol II degradation, we performed shRNA knockdown experiments for each BTB to assess their requirement for Pol II destabilization upon SPT5 depletion. To assess knockdown efficiency, we initially attempted to quantify ARMC5 protein on Western blots using commercially available antibodies, but none worked for our cell line. We therefore performed reverse transcription-quantitative polymerase chain reaction (RT-qPCR) and RNA-sequencing (RNA-seq) in SPT5-AID cells with ARMC5 knockdown and confirmed that ARMC5 mRNA was significantly depleted compared to nontargeting (NT) knockdown (Fig. 1F and fig. S1, D and E). Further analysis of RNA-seq data showed that the differentially expressed genes in ARMC5-depleted relative to NT shRNA–treated cells are enriched for pathways including immune response signaling (fig. S1F). SPT5-depleted Pol II was significantly stabilized by ARMC5 or CUL3 knockdown but not by treatment with NT shRNA (Fig. 1G and fig. S1G). To examine whether ARMC5 targets chromatin-bound Pol II, we separated chromatin pellets from soluble nuclear fractions. The chromatin pellet fractions, which enrich phosphorylated forms of Pol II, showed substantial stabilization of SPT5-depleted Pol II by ARMC5 knockdown (Fig. 1H). Stability of unphosphorylated, free Pol II observed in nuclear fractions was also partially restored (Fig. 1H). This result supports a role for the CUL3-ARMC5 pathway in specific elimination of chromatin-bound (but SPT5-less) Pol II beyond regulation of free Pol II stability. Unlike ARMC5, neither BTBD2 nor KLHL7 shRNA knockdown restored Pol II stability upon SPT5 loss (fig. S1, H to J). Given the role for CUL4 in Pol II degradation in response to DNA damage (*20*), we also investigated the possible involvement of CUL4 in Pol II degradation upon SPT5 loss. When we depleted DNA damage–binding protein 1 (DDB1), a core component of the CUL4-DDB1 E3 ubiquitin ligase complex, no appreciable restoration of Pol II stability was observed (fig. S1K). Collectively, these results identify ARMC5 as a CUL3 adaptor required for the specific CUL3-dependent degradation of Pol II upon SPT5 loss.

### ARMC5 loss restores abundance but not function of SPT5-depleted Pol II

**Fig. 2.**
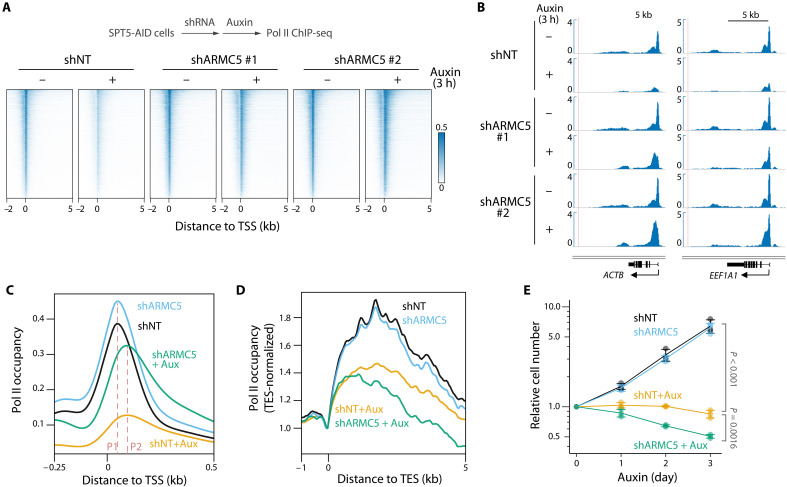
Defective Pol II transcription in the absence of SPT5 is partially rescued by ARMC5 loss. (**A** to **D**) TSS-centered signal heatmaps (A), representative signal tracks (B), and plots of average signal centered on TSS (C) and TES (D) for Pol II ChIP-seq in SPT5-AID cells treated with ARMC5 shRNA followed by auxin (500 μM, 3 hours). *N* = 6481. In (C), P1: the first pause site; P2: the second pause site. In (D), signal was normalized to signal at TES. (**E**) Growth curve for SPT5-AID cells treated with ARMC5 shRNA followed by auxin (500 μM). *N* = 3, Tukey’s test. Error bars, SD.

Given the multiple regulatory roles for SPT5 in transcriptional elongation, we examined whether ARMC5 depletion could rescue the elongation defects observed upon SPT5 loss by performing ChIP-seq experiments for total Pol II in SPT5-AID cells treated with ARMC5 shRNA followed by auxin. Consistent with our Western blot analysis (Fig. 1, G and H), Pol II signal was largely restored genome-wide in response to ARMC5 knockdown followed by SPT5 depletion (Fig. 2, A and B). However, we observed distinct patterns of Pol II signal distribution in the rescue compared to untreated conditions (Fig. 2B). Genome-wide examination of Pol II occupancy at promoter-proximal regions near transcription start sites (TSSs) revealed that the average location of Pol II pause sites (the location of the peak in amplitude of the average promoter-proximal Pol II signal distribution, relative to the location of TSS) was not altered by single ARMC5 knockdown, although the amplitude of average Pol II signal was slightly increased (Fig. 2C). As expected, average Pol II signal was strongly decreased by SPT5 depletion and the average location of Pol II pause sites was shifted downstream to a second pause site (Fig. 2C and fig. S2A). A substantial increase in Pol II signal was observed relative to the SPT5 depletion–only condition when both ARMC5 and SPT5 were depleted. However, even in this rescue condition, Pol II remained paused at the second pause site (Fig. 2C and fig. S2A). At the 3′ end of genes, single ARMC5 knockdown did not affect the genome-wide average distribution of Pol II signal around transcription end sites (TESs) (Fig. 2, B and D, and fig. S2B). Genome-wide Pol II signal was diminished by SPT5 depletion downstream of TES, and similarly to observations at TSS, this was not rescued by knockdown of ARMC5 (Fig. 2D and fig. S2B). Collectively, these results suggest that ARMC5 depletion specifically restores abundance of promoter-proximal Pol II but does not rescue elongation defects caused by SPT5 loss.

As defects in transcriptional elongation are associated with severe growth defects (*6*, *8*, *14*), we monitored the growth of SPT5-AID cells treated with ARMC5 shRNA followed by auxin using crystal violet staining (Fig. 2E and fig. S2C). SPT5-AID cells with single ARMC5 knockdown grew normally (Fig. 2E), despite changes in gene expression (fig. S1E). As expected, SPT5 depletion immediately and completely stopped cell growth (Fig. 2E). Cells with double depletion exhibited a similarly profound growth defect (Fig. 2E). These observations suggest that elongating Pol II complexes that lack SPT5 are truly defective even when stabilized, to the extent that they fail to support the transcriptional demands of cell growth.

### The BTB domain of ARMC5 is required for CUL3 to target SPT5-depleted Pol II

**Fig. 3.**
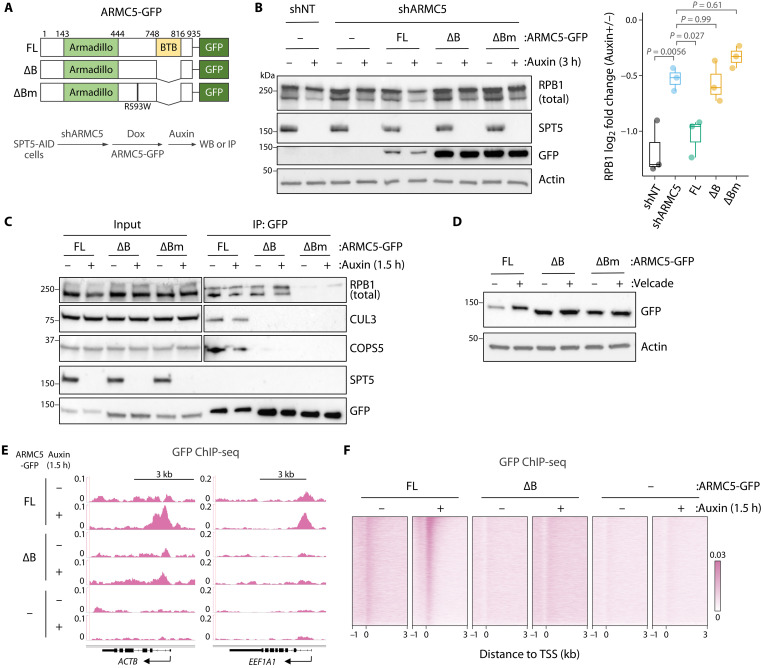
The BTB domain of ARMC5 is required for CUL3 to target SPT5-depleted Pol II. (**A**) Schematic of GFP-tagged ARMC5 constructs. (**B**) Western blot analysis of whole-cell extracts from ARMC5-GFP construct–expressing SPT5-AID cells treated with auxin (500 μM, 3 hours). Box plot shows fold change of RPB1 protein upon SPT5 loss. *N* = 3, Tukey’s test. (**C**) Western blot analysis of ARMC5-GFP IP showing interaction with Pol II and CUL3 in ARMC5-GFP construct–expressing SPT5-AID cells treated with auxin (500 μM, 1.5 hours). (**D**) Stability of ARMC5-GFP protein with or without Velcade treatment (1 μM, 6 hours). (**E** and **F**) Representative signal tracks (E) and TSS-centered signal heatmaps (F) for GFP ChIP-seq in ARMC5-GFP construct–expressing SPT5-AID cells treated with auxin (500 μM, 1.5 hours). *N* = 6481.

The ARMC5 protein contains a BTB domain that is structurally conserved among CUL3 adaptors and physically interacts with CUL3 (*24*). To examine the role for the ARMC5 BTB domain in Pol II degradation upon SPT5 loss, we generated GFP-tagged ARMC5 constructs that allow for doxycycline-inducible expression in ARMC5-depleted cells (Fig. 3A and fig. S3A). The full-length (FL) ARMC5-GFP expression led to the Pol II degradation in response to SPT5 depletion (Fig. 3B). GFP IP revealed that FL ARMC5-GFP interacts with Pol II, CUL3, and the CUL3-associated constitutive photomorphogenesis 9 signalosome (CSN) component COPS5 (Fig. 3C and fig. S3B). These results indicate that the GFP-tagged ARMC5 construct recapitulates the normal function of endogenous ARMC5. The weak interaction with Pol II (fig. S3B) might be due to other substrates that could occupy most ARMC5 proteins. Interaction with CUL3 was also weak (fig. S3B), which can be explained by dynamic nature of adaptor exchange (*24*). Notably, SPT5 was not detected in ARMC5-GFP coimmunoprecipitants (Fig. 3C). Similarly, CUL3 and ARMC5 were not detected in SPT5 coimmunoprecipitants (fig. S3C). Thus, it is likely that ARMC5-CUL3 preferentially interact with Pol II that lacks SPT5 compared to SPT5-bound Pol II. In contrast to FL, the ARMC5-GFP construct with BTB domain deletion (∆B) failed to mediate Pol II degradation and did not interact with CUL3 or COPS5 (Fig. 3, B and C). These results indicate that ARMC5 BTB domain facilitates formation of the Pol II–recognizant CUL3 complex required for degradation of SPT5-depleted Pol II. On the other hand, BTB deletion had little effect on ARMC5 interaction with Pol II (Fig. 3C). It was previously shown that this interaction is abolished by a single mutation, R593W, derived from an adrenal hyperplasia patient (*27*). Consistent with this finding, addition of the R593W mutation to ∆B (∆Bm) abolished interaction between ARMC5 and Pol II (Fig. 3C) without resulting in a further Pol II degradation defect (Fig. 3B). Neither ∆B nor ∆Bm could mediate Pol II degradation upon SPT5 loss (Fig. 3B). We noted lower protein levels for the FL relative to the ∆B and ∆Bm constructs (Fig. 3B and fig. S3A), consistent with BTB domain–dependent autodegradation (*29*). The FL construct was efficiently stabilized by proteasome inhibitor Velcade/bortezomib, while stability of the ∆B and ∆Bm constructs was largely unchanged (Fig. 3D).

Given that chromatin-bound, promoter-proximal Pol II is targeted for degradation upon SPT5 loss (*14*), we examined the chromatin localization of ARMC5 in auxin-treated SPT5-AID cells. On the basis of the Pol II abundance in a time-course auxin treatment, we chose to use the 1.5-hour time point, at which no SPT5 is detectable in whole-cell lysates (Fig. 1G) but most Pol II remains on chromatin (fig. S4, A to C). We reasoned that this remaining Pol II would be enriched for ARMC5 interaction. We performed GFP ChIP-seq in SPT5-AID cells expressing ARMC5-GFP constructs and found that ARMC5-GFP (FL) signal is substantially increased at promoter-proximal regions in response to SPT5 loss (Fig. 3, E and F). In the presence of SPT5, a weak signal of ARMC5 (FL) was detected in cells expressing ARMC5-GFP (FL) compared to the background signal observed in the no-GFP control (Fig. 3, E and F). Deletion of the BTB domain (∆B) abolished recruitment of ARMC5 to promoter-proximal regions (Fig. 3, E and F). This stands in contrast to the finding that the ARMC5 BTB is dispensable for interaction with bulk Pol II (Fig. 3C).

### CDK9 activity is required for ARMC5 interaction with promoter-proximal Pol II

**Fig. 4.**
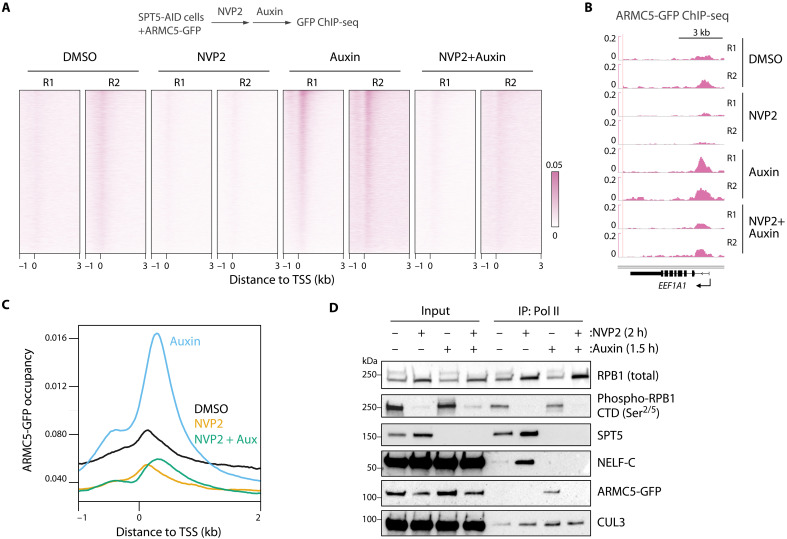
ARMC5 interaction with Pol II requires CDK9 activity. (**A** to **C**) TSS-centered signal heatmaps (A), representative signal tracks (B), and TSS-centered average signal plot (C) for GFP ChIP-seq in ARMC5-GFP–expressing SPT5-AID cells treated with NVP-2 (250 nM, 2 hours) followed by auxin (500 μM, 1.5 hours). In (A) and (B), data from two biological replicates (R1, R2) are shown. *N* = 6841. (**D**) Western blot analysis of Pol II IP in SPT5-AID cells, treated as in (A) to (C).

The fact that CDK9 inhibition blocks degradation of SPT5-depleted Pol II suggests that CDK9 could regulate ARMC5 recruitment to chromatin-bound Pol II. To examine this possibility, we treated ARMC5-GFP–expressing SPT5-AID cells with the CDK9 inhibitor NVP-2 followed by auxin and performed GFP ChIP-seq to assess ARMC5 chromatin occupancy. Compared to the high levels of signal observed upon single auxin treatment, ARMC5 ChIP-seq signal was largely decreased by double treatment with NVP-2 and auxin (Fig. 4, A to C). Thus, CDK9 kinase activity appears to regulate ARMC5 recruitment to promoter-proximal Pol II, in good agreement with our initial MS results (Fig. 1D). To verify this CDK9-dependent Pol II–ARMC5 interaction, we performed IP for Pol II in ARMC5-GFP–expressing SPT5-AID cells treated with NVP-2 followed by auxin. As expected, single NVP-2 treatment led to loss of phosphorylation at Pol II C-terminal domain (CTD) serine-2/serine-5 (Fig. 4D), which are the direct targets of CDK9 (*30*, *31*). While no appreciable Pol II–ARMC5 interaction was observed in the untreated condition, the Pol II–ARMC5 interaction was clearly evident upon SPT5 depletion (Fig. 4D). The Pol II–CUL3 interaction was also enhanced by SPT5 depletion, suggesting a recruitment of the CUL3-ARMC5 ubiquitin ligase complex to SPT5-depleted Pol II. NVP-2 pretreatment abolished Pol II interaction with ARMC5 and diminished its interaction with CUL3 (Fig. 4D). Partial loss of CUL3 in this condition (Fig. 4D) implies that Pol II immunoprecipitants might contain other CUL3 substrates. Together, these results indicate that CDK9 activity is required for ARMC5 interaction with promoter-proximal Pol II, leading to an efficient elimination of SPT5-depleted Pol II specifically at promoter-proximal regions.

**Fig. 5.**
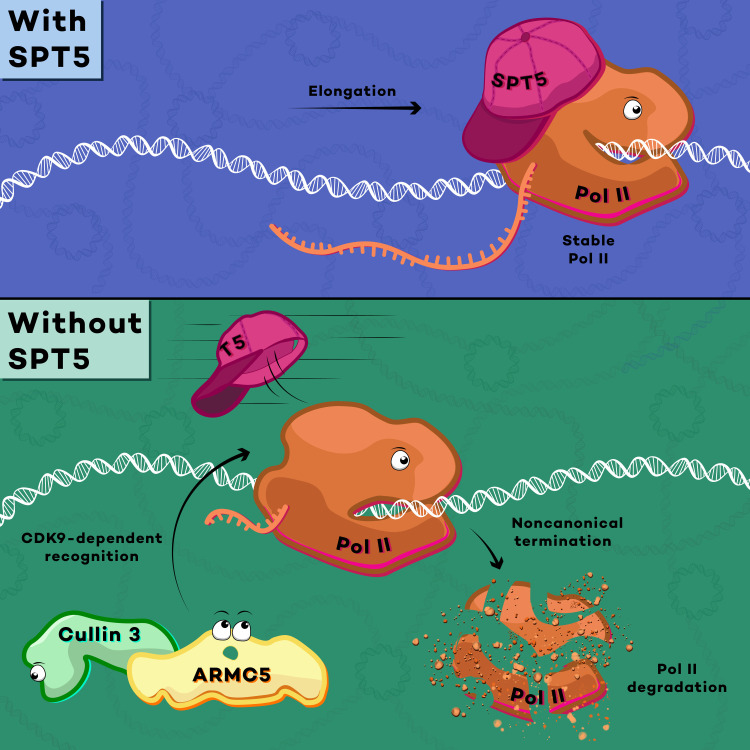
Model for noncanonical termination of promoter-proximal Pol II transcription. SPT5 stabilizes Pol II elongation complex (**top**). Upon SPT5 loss (**bottom**), the ARMC5-CUL3 ubiquitin ligase complex recognizes promoter-proximal Pol II in a CDK9-dependent manner, leading to degradation-dependent transcription termination.

## DISCUSSION

Following our recent identification of the CUL3-dependent Pol II degradation pathway (*14*), it remained unclear how CUL3 recognizes promoter-proximal Pol II and how CDK9 regulates Pol II degradation. Here, our results provide molecular details of the mechanisms that govern degradation of promoter-proximal Pol II: We show that Pol II lacking SPT5 is recognized by the CUL3 adaptor ARMC5 and that ARMC5 interaction with Pol II requires the kinase activity of CDK9 (Fig. 5). Before this study, loss-of-function heterozygous mutations affecting ARMC5 were detected in primary bilateral macronodular adrenal hyperplasia (*28*) and ARMC5 was found to regulate the abundance of bulk Pol II via direct interaction (*26*, *27*). We demonstrate that ARMC5 functions to regulate stability of chromatin-bound, elongating Pol II, in addition to chromatin-free Pol II that is either on the way to its next round of transcription or has just been synthesized by the ribosome. Our biochemical results indicate that ARMC5 targets SPT5-depleted chromatin-bound Pol II only when, and therefore where, CDK9 is active. We previously observed CUL3 occupancy at promoter-proximal regions even in the presence of SPT5. It is likely that ARMC5 searches for Pol II that both lacks SPT5 and contains high CDK9 activity to specifically find defective elongation complex on chromatin. By contrast, it remains largely unclear how degradation of free Pol II is regulated. It is tempting to speculate that CUL3-ARMC5 targets free Pol II that accidently contains high CDK9 activity, which will disturb pause-release regulation if the next transcription cycle is initiated. We note that while our manuscript was under review, Blears *et al.* (*32*) independently reported ARMC5-dependent removal of promoter-proximal Pol II.

Following recognition by CUL3-ARMC5, promoter-proximal Pol II that lacks SPT5 undergoes VCP/p97-dependent unfolding (*14*), which halts transcript elongation. Given our previous finding that SPT5 is required for Pol II processivity and our present findings of defective elongation and a profound growth defect upon SPT5 depletion, neither of which is rescued by restoration of Pol II levels, we consider elongation complexes that lack SPT5 to be truly “defective.” Further, we propose that AMRC5-dependent Pol II degradation constitutes a noncanonical “termination” pathway used to eliminate defective elongation complexes from promoter-proximal regions. This CUL3-dependent pathway stands in contrast to canonical termination as well as noncanonical CUL4-dependent pathways used to rectify elongation complexes stalled at DNA lesions. The canonical termination pathway at the 3′ end of genes uses a process that requires cleavage of nascent RNA around polyadenylation signal (PAS) and the subsequent degradation of uncapped transcripts that remain attached to Pol II (*33*). The CUL4-dependent degradation in response to DNA damage is initiated by CSB that interacts with the Pol II interface adjacent to the upstream DNA, which is also used for interaction with SPT5 (*20*). Previous structural studies suggested that replacement of SPT5 with CSB upon DNA damage is required for the CUL4-dependent degradation (*22*, *34*). Future studies will be needed to identify mechanisms that determine the use of specific cullin ubiquitin ligase and to characterize a context-specific role for SPT5 in Pol II degradation.

Loading of SPT5 onto Pol II is one of the earliest transcriptional elongation events, occurring immediately after the establishment of preinitiation complexes at core promoters (*35*). Dissociation of initiation factors including TFIIE and TFIIH and recruitment of SPT5 take place simultaneously, as mutual binding of both TFIIE and SPT5 to Pol II is disallowed by steric clash (*36*). The TFIIH-associated CDK7 kinase functions to regulate this TFIIE-SPT5 swap (*37*). The transcription factor MYC was also shown to recruit SPT5 via direct interaction (*38*). In the case of dysregulation or dysfunction in these processes, a failure to load SPT5 onto Pol II may be recognized by ARMC5 and rectified by CUL3-mediated degradation of the defective Pol II.

We have provided evidence for unique features of a CUL3-dependent Pol II degradation pathway in human cells. However, many questions remain. What is the critical substrate of CDK9 phosphorylation required for ARMC5 interaction with Pol II? Which lysine residues are ubiquitinated by the CUL3-ARMC5 ligase complex? Moreover, while the SPT5 protein is highly conserved in eukaryotes and broadly conserved across all three domains of life, the pervasiveness of CUL3-dependent degradation pathway and the potential for alternative mechanisms to detect SPT5-deficient Pol II is unclear. It will be important for future studies to address these questions to understand the mechanisms that regulate degradation-dependent transcriptional termination.

## MATERIALS AND METHODS

### Cell culture

The human DLD-1 SPT5-AID cell line was generated in our previous study using AtAFB2 (*14*, *39*). Cells used were cultured in Dulbecco’s modified Eagle’s medium (DMEM) (Sigma, catalog no. D6429) supplemented with 10% fetal bovine serum (FBS) (Sigma, catalog no. F2442) and 1% penicillin-streptomycin (Gibco, catalog no. 15140122) at 37°C in the presence of 5% CO_2_. Auxin (Abcam, catalog no. ab146403) was directly added to culture for SPT5-AID depletion. NVP2 (MedChemExpress, catalog no. HY-12214A) and Velcade/bortezomib (Cayman, catalog no. 10008822) were used to inhibit CDK9 and proteasome, respectively. Cells used in this study tested negative for mycoplasma using PCR-based universal mycoplasma detection kit [American Type Culture Collection (ATCC), catalog no. 30-1012K].

### GFP-tagged ARMC5 and CUL3

The amino acid sequences of the canonical isoforms of ARMC5 (UniProt, Q96C12) and CUL3 (UniProt, Q13618) were used to design the constructs. DNA fragments for ARMC5 and GFP were obtained from Twist Biosciences and inserted into pCW57-MCS1-P2A-MCS2 (Blast) (Addgene, catalog no. 80921) using NEBuilder HiFi DNA assembly (NEB, catalog no. M5520A). DNA fragments for CUL3 and GFP were assembled with the pCW57-MCS1-P2A-MCS2 (Puro) (Addgene, catalog no. 71782). Plasmid sequences were determined using Plasmidsaurus long-read sequencing service or ACGT Inc. service provided by Northwestern University Sanger sequencing core. Lentivirus production and infection were performed as described below. The infected cells were cultured in the presence of blasticidin (5 μg/ml; Gibco, catalog no. A1113903) or puromycin (2 μg/ml) for selection. Doxycycline (1 μg/ml; Sigma, catalog no. D9891-1G) was used to induce expression of GFP-tagged genes.

### Growth assay

Cells (35,000) were seeded onto six-well plates and treated with 500 μM auxin on the next day (day 0). For each time point, cells were fixed in 4% formaldehyde (Sigma, catalog no. 252549) in phosphate-buffered saline (PBS) for 20 min at room temperature. The fixed cells were stained by crystal violet solution (Sigma, catalog no. HT90132). The stain was dissolved in 10% acetic acid. Absorbance at 590 nm was measured by Spark microplate reader (Tecan).

### shRNA knockdown

GIPZ lentiviral shRNA constructs against CUL3 (RHS4430-200161789), ARMC5 (RHS4430-200295250, RHS4430-200297132), BTBD2 (RHS4430-200226126, RHS4430-200226529), KLHL7 (RHS4430-200213137, RHS4430-200264380), and DDB1 (RHS4430-200187615, RHS4430-200223811) from Horizon Discovery were used. NT shRNA (RHS4346) was used as a negative control.

GIPZ shRNA, psPAX2, and pMD2.G plasmids were cotransfected into 293T cells (ATCC, catalog no. CRL-3216) using Lipofectamine 3000 (Invitrogen, catalog no. L3000015) for shRNA lentiviral production. Lentivirus in supernatant was precipitated using Lenti-X concentrator (Takara, catalog no. 631232) and resuspended in serum-free DMEM. The concentrated virus was snap-frozen in liquid nitrogen and stored at −80°C. SPT5-AID cells were infected with shRNA lentivirus in the presence of polybrene (8 μg/ml; Sigma, catalog no. TR-1003). The infected cells were selected in the presence of puromycin (2 μg/ml; Gibco, catalog no. A11138-03).

### Western blotting

Whole-cell extracts were prepared by direct cell lysis in 2× Laemmli buffer and boiled for 8 min. Proteins were separated in 4 to 20% Mini-PROTEAN TGX Precast Proteins Gels (Bio-Rad, catalog nos. 4561094 and 4561096) and transferred to nitrocellulose membranes. Membranes were stained with 0.1% Ponceau S in 5% acetic acid. Following blocking with 5% milk/tris-buffered saline with Tween 20 (TBST), membranes were incubated with primary antibodies diluted in 5% bovine serum albumin (BSA)/TBST or 1% milk/TBST at 4°C overnight. After washes with TBST, membranes were incubated with secondary horseradish peroxidase (HRP)–linked antibodies diluted in 1% milk/TBST for 1 hour at room temperature. Immobilon Crescendo Western HRP substrate (Millipore, catalog no. WBLUR0500) was used for chemiluminescence. Signal was detected on ChemiDoc imaging system (Bio-Rad). Antibodies used in this study are listed in table S2.

### Chromatin/nuclear fractionation

Cells were lysed in hypotonic nuclei EZ lysis buffer (Sigma, catalog no. NUC101) supplemented with protease/phosphatase inhibitors (Thermo Fisher Scientific, catalog nos. A32965 and A32957) for 5 min on ice. Following centrifugation (9800*g*, 4°C, 3 min), nuclei were incubated in nuclei EZ lysis buffer supplemented with 500 mM NaCl and protease/phosphatase inhibitors for 15 min on ice. Samples were centrifuged (20,000*g*, 4°C, 3 min) to separate soluble nuclear fraction from insoluble chromatin pellet. Fractions were mixed with Laemmli buffer and boiled. Chromatin fraction was passed through 27-gauge needles to reduce viscosity. Each fraction was analyzed in SDS-PAGE (polyacrylamide gel electrophoresis)/Western blotting.

### ChIP sequencing

Twenty-five million cells were fixed in 1% (w/v) formaldehyde (Thermo Fisher Scientific, catalog no. 28908)–containing PBS for 10 min at room temperature. Glycine (250 mM) was added to quench for 5 min at room temperature. Following washing with PBS, fixed cells were collected using scrapers, snap-frozen, and stored at −80°C.

Fixed cells were lysed in lysis buffer 1 [50 mM Hepes (pH 7.5), 140 mM NaCl, 1 mM EDTA, 10% glycerol, 0.5% IGEPAL CA-630, 0.25% Triton X-100, protease inhibitor (Sigma, catalog no. P8340)] for 10 min at 4°C. Samples were centrifuged at 1350*g* for 3 min at 4°C, and pellets were washed in lysis buffer 2 [10 mM tris-HCl (pH 8.0), 200 mM NaCl, 1 mM EDTA, 0.5 mM EGTA, protease inhibitor] for 10 min at 4°C. Pellets were then resuspended in lysis buffer 3 [10 mM tris-HCl (pH 8.0), 1 mM EDTA, 0.1% SDS, protease inhibitor] and solubilized on E220 focused-ultrasonicator (Covaris) using milliTUBE 1 ml AFA Fiber tube (Covaris, catalog no. 520130) with 10% duty cycle, 140 peak intensity power, and 200 cycles per burst for 600 s at 4°C.

Following centrifugation at 20,000*g* for 15 min at 4°C, supernatants were mixed with spike-in chromatin and one-ninth volume of 10× ChIP dilution buffer (10% Triton X-100, 1 M NaCl, 1% sodium deoxycholate, 5% N-lauroylsarcosine, 5 mM EGTA). For spike-in chromatin, mouse embryonic fibroblasts or *Drosophila* S2 fly cells were used. Chromatin samples were incubated with antibodies overnight at 4°C. For GFP ChIP, fly spike-in chromatin was captured by spike-in normalization antibody (Active Motif, catalog no. 61686). Dynabeads Protein G was added to the samples and incubated for 4 hours at 4°C. Beads were washed four times with radioimmunoprecipitation assay (RIPA) buffer [50 mM Hepes (pH 7.5), 500 mM LiCl, 1 mM EDTA, 1% IGEPAL CA-630, 0.7% sodium deoxycholate] and once with TEN buffer [tris-EDTA (TE) + 50 mM NaCl], followed by elution in elution buffer [50 mM tris-HCl (pH 8.0), 10 mM EDTA, 1% SDS] supplemented with proteinase K (400 μg/ml; Sigma, catalog no. 3115887001) overnight at 65°C on themomixer at 1200 rpm. DNA was purified using QIAquick PCR Purification Kit (Qiagen, catalog no. 28106). KAPA HyperPrep Kit (Roche, catalog no. KR0961) was used to prepare libraries for sequencing on NovaSeq 6000 (Illumina) or Nextseq 2000 (Illumina).

ChIP-seq data analysis: Raw reads were quality-trimmed using trimmomatic 0.39 (*40*) and aligned to a concatenated genome assembly (hg38 + mm10, or hg38 + dm6) using bowtie 2.4.5 with --sensitive option (*41*). Reads with mapping quality (MAPQ) ≥30 were used to generate bigwig coverage files. Read counts were normalized to total mapped reads in spike-in genome. Heatmaps and average profiles were generated using deepTools 3.5.1 (*42*). Genes used in ChIP-seq analysis were selected, and TSSs for these genes were identified de novo based on our previous PRO-cap data in DLD-1 cells (*8*, *14*). Sequencing data from Gene Expression Omnibus (GEO): GSE168827 were reanalyzed and shown in Fig. 1 (B and C).

### RNA sequencing

RNeasy Mini kit and QIAshredder (Qiagen, catalog nos. 74106 and 79656) were used to extract total RNA. On-column deoxyribonuclease (DNase) digestion was performed to eliminate genomic DNA. cDNA synthesis and library preparation were performed using NEBNext Poly(A) mRNA magnetic isolation module (NEB, catalog no. E7490) and NEBNext Ultra II RNA library prep kit for Illumina (NEB, catalog no. E7770). Libraries were sequenced on Nextseq 2000 (Illumina).

RNA-seq data analysis: After quality-trimming using cutadapt 4.2 (*43*), reads were applied to transcript quantification using salmon 1.9.0 with --gcBias option (*44*) and summarization using tximeta (*45*). Genes with ≥10 counts in at least three samples were retained. Differential expression analysis was performed using DESeq2 1.44.0 (*46*). Differences with adjusted *P* value <0.1 were considered significant. For visualization, log_2_ fold change shrinkage was performed using ashr (*47*). GSEA (gene set enrichment analysis) was performed using ReactomePA (*48*).

### Reverse transcription-qPCR

Extraction of total RNA and elimination of genomic DNA were carried out as mentioned above. Reverse transcription and real-time qPCRs were performed using Luna universal one-step RT-qPCR kit (NEB, catalog no. E3005S) on CFX Connect real-time system (Bio-Rad). PrimeTime predesigned qPCR primers for ARMC5 (Hs.PT.58.21355090.g), BTBD2 (Hs.PT.58.21450467), and Actin (Hs.PT.39a.22214847) from IDT were used to quantify each target transcript.

### Immunoprecipitation

Pol II IP was performed using nuclei, and SPT5/GFP IP was performed using whole-cell extracts. For nuclei isolation, 60 million cells were first treated with 250 nM NVP2 for 2 hours followed by treatment with 500 μM auxin for 3 hours. Cell culture plates were washed with cold PBS twice at 4°C. Lysis buffer [10 mM tris-HCl (pH 7.4), 10 mM KCl, 1.5 mM MgCl_2_, 12% (w/v) sucrose, 10% (v/v) glycerol, 0.2% Triton X-100, protease inhibitor EDTA-free (Thermo Fisher Scientific, catalog no. A32965), phosphatase inhibitor EDTA-free (Thermo Fisher Scientific, catalog no. A32957), 0.5 mM dithiothreitol (DTT)] was added to the cells and incubated for 5 min. Cell lysates were collected using scrapers and centrifuged at 800*g* for 5 min at 4°C. Pellets were resuspended in the lysis buffer again. Sucrose cushion buffer [10 mM tris-HCl (pH 7.4), 10 mM KCl, 1.5 mM MgCl_2_, 30% (w/v) sucrose, 0.5 mM DTT] was added as a layer beneath the resuspended pellet. Samples were centrifuged at 800*g* for 20 min at 4°C. Pellets were resuspended in freezing buffer [10 mM tris-HCl (pH 7.4), 10 mM KCl, 1.5 mM MgCl_2_, 40% (v/v) glycerol, 0.5 mM DTT], snap-frozen in liquid nitrogen, and stored at −80°C. For whole-cell extracts, 20 million cells were trypsinized, washed with PBS, snap-frozen, and stored at −80°C.

Chromatin digestion: For Pol II IP, nuclei pellets were resuspended in benzonase-containing chromatin digestion buffer [20 mM tris-HCl (pH 7.4), 150 mM NaCl, 1.5 mM MgCl_2_, 10% glycerol, 0.05% IGEPAL, protease inhibitor EDTA-free, phosphatase inhibitor EDTA-free, 0.5 mM DTT, benzonase (1 U/μl; Sigma, catalog no. E1014)] and incubated for 1 hour at 4°C with rotation. Samples were centrifuged at 20,000*g* for 5 min at 4°C. Supernatants were collected in 5-ml tubes. Pellets were resuspended in chromatin-2 buffer (chromatin digestion buffer with 500 mM NaCl and 3 mM EDTA) and incubated at 4°C with rotation for 30 min and then diluted in chromatin-3 buffer (chromatin digestion buffer with 3 mM EDTA) to lower the NaCl concentration to 185 mM. Lysates were centrifuged at 20,000*g* for 5 min at 4°C, and supernatants were pooled with the first supernatant. For SPT5/GFP chromatin digestion, cell pellets were resuspended in benzonase-containing chromatin digestion buffer and incubated for 1 hour at 4°C with rotation. EDTA (1 mM) was added to each sample, and samples were centrifuged at 20,000*g* for 5 min at 4°C. Supernatants were used for IP.

IP: For Pol II or SPT5 IP, antibody-bound Dynabeads Protein G (Invitrogen, catalog no. 10004D) was incubated with lysates for 90 min at 4°C. For GFP IP, GFP-trap magnetic agarose beads (Proteintech, catalog no. gtma) were added to the lysates and incubated at 4°C for 1 hour. Beads were washed with washing buffer [20 mM tris-HCl (pH 7.4), 150 mM NaCl, 1.5 mM MgCl_2_, 10% glycerol, 0.05% IGEPAL, 0.5 mM DTT] three times and with 100 mM ammonium bicarbonate twice. Pol II and SPT5 immunoprecipitants were eluted in mild elution buffer [0.1% (w/v) SDS, 50 mM tris-HCl (pH 8.0), 1 mM EDTA]. GFP immunoprecipitants were eluted in acidic elution buffer (0.2 M glycine, pH 2.5) followed by neutralization with 1 M tris-HCl (pH 10.4). Eluates were snap-frozen in liquid nitrogen and stored at −80°C.

### Mass spectrometry

Filter-aided sample preparation methods—Orbitrap Exploris data-independent acquisition (DIA): Protein samples were reduced, alkylated, and digested using filter-aided sample preparation (*49*) with sequencing-grade modified porcine trypsin (Promega). Tryptic peptides were then separated by reversed-phase XSelect CSH C18 2.5 μm resin (Waters) on an in-line 150 × 0.075 mm column using an UltiMate 3000 RSLCnano system (Thermo Fisher Scientific). Peptides were eluted using a 60-min gradient from 98:2 to 65:35 buffer A:B ratio. Eluted peptides were ionized by electrospray (2.2 kV) followed by MS analysis on an Orbitrap Exploris 480 mass spectrometer (Thermo Fisher Scientific). To assemble a chromatogram library, six gas-phase fractions were acquired on the Orbitrap Exploris with 4 mass/charge ratio (*m*/*z*) DIA spectra (4 *m*/*z* precursor isolation windows at 30,000 resolution, normalized automatic gain control (AGC) target 100%, maximum inject time 66 ms) using a staggered window pattern from narrow mass ranges with optimized window placements. Precursor spectra were acquired after each DIA duty cycle, spanning the *m*/*z* range of the gas-phase fraction (i.e., 496 to 602 *m*/*z*, 60,000 resolution, normalized AGC target 100%, maximum injection time 50 ms). For wide-window acquisitions, the Orbitrap Exploris was configured to acquire a precursor scan (385 to 1015 *m*/*z*, 60,000 resolution, normalized AGC target 100%, maximum injection time 50 ms) followed by 50× 12 *m*/*z* DIA spectra (12 *m*/*z* precursor isolation windows at 15,000 resolution, normalized AGC target 100%, maximum injection time 33 ms) using a staggered window pattern with optimized window placements. Precursor spectra were acquired after each DIA duty cycle. Buffer A = 0.1% formic acid, 0.5% acetonitrile; Buffer B = 0.1% formic acid, 99.9% acetonitrile.

Following data acquisition, data were searched using an empirically corrected library against the UniProt *Homo sapiens* database (January 2023) and a quantitative analysis was performed to obtain a comprehensive proteomic profile. Proteins were identified and quantified using EncyclopeDIA (*50*) and visualized with Scaffold DIA (version 3.3.1) using 1% false discovery thresholds at both the protein and peptide level. Protein MS2 exclusive intensity values were assessed for quality using ProteiNorm (*51*). The data for GFP-CUL3 IP were normalized using Cyclic Loess (*52*) and analyzed using proteoDA to perform statistical analysis using Linear Models for Microarray Data (limma) with empirical Bayes (eBayes) smoothing to the standard errors (*52*, *53*). Proteins with a false discovery rate (FDR)–adjusted *P* value <0.01 and a fold change >2 were considered significant. Protein annotation for SKP1/BTB/POZ domain superfamily (InterPro, IPR011333) was used to search for the BTB proteins. For Pol II IP data, outlier samples with many missing values were discarded based on quality assessment using ProteiNorm (*51*). Using proteoDA (*53*), proteins found in >0.66 proportion of samples within each group were retained and contaminants were filtered out. Log_2_-transformed protein intensities were normalized to Pol II–specific core subunits, according to global standard normalization method in MSstats (*54*). Following imputation using QRILC (*55*), protein intensities for BTB, CUL3, DSIF, and NELF proteins were applied for clustering with row-wise scaling using pheatmap (*56*).

### Statistical analysis

Analysis of variance (ANOVA) and post hoc Tukey HSD (honestly significant difference) tests were used for multiple pairwise comparisons in growth assay, quantitative Western blotting, and RT-qPCR data to control family-wise error rate. Wald test in DESeq2 package was used for RNA-seq analysis. Limma moderated *t* test was used for GFP-CUL3 IP-MS data analysis. Benjamini-Hochberg procedure was used for Wald and limma tests to control FDR. *N*, number of biological replicates or number of genes tested.
