# Supplementary material for: SPT5 regulates RNA polymerase II stability via Cullin 3–ARMC5 recognition
Source: Sci Adv. 2025 Jan 24;11(4):eadt5885. doi: 10.1126/sciadv.adt5885 (PMC11758996; doi:10.1126/sciadv.adt5885)
Supplement: Supplementary file 1 — Figs. S1 to S4 Legend for table S1 Table S2 [file sciadv.adt5885_sm.pdf]

Science Advances
AAAS

Supplementary Materials for

SPT5 regulates RNA polymerase II stability via Cullin 3–ARMC5 recognition

Yuki Aoi *et al.*

Corresponding author: Ali Shilatifard, ash@northwestern.edu; Yuki Aoi, yuki.aoi@northwestern.edu

Sci. Adv. **11**, eadt5885 (2025)
DOI: 10.1126/sciadv.adt5885

The PDF file includes:

Figs. S1 to S4
Legend for table S1
Table S2

Other Supplementary Material for this manuscript includes the following:

Table S1

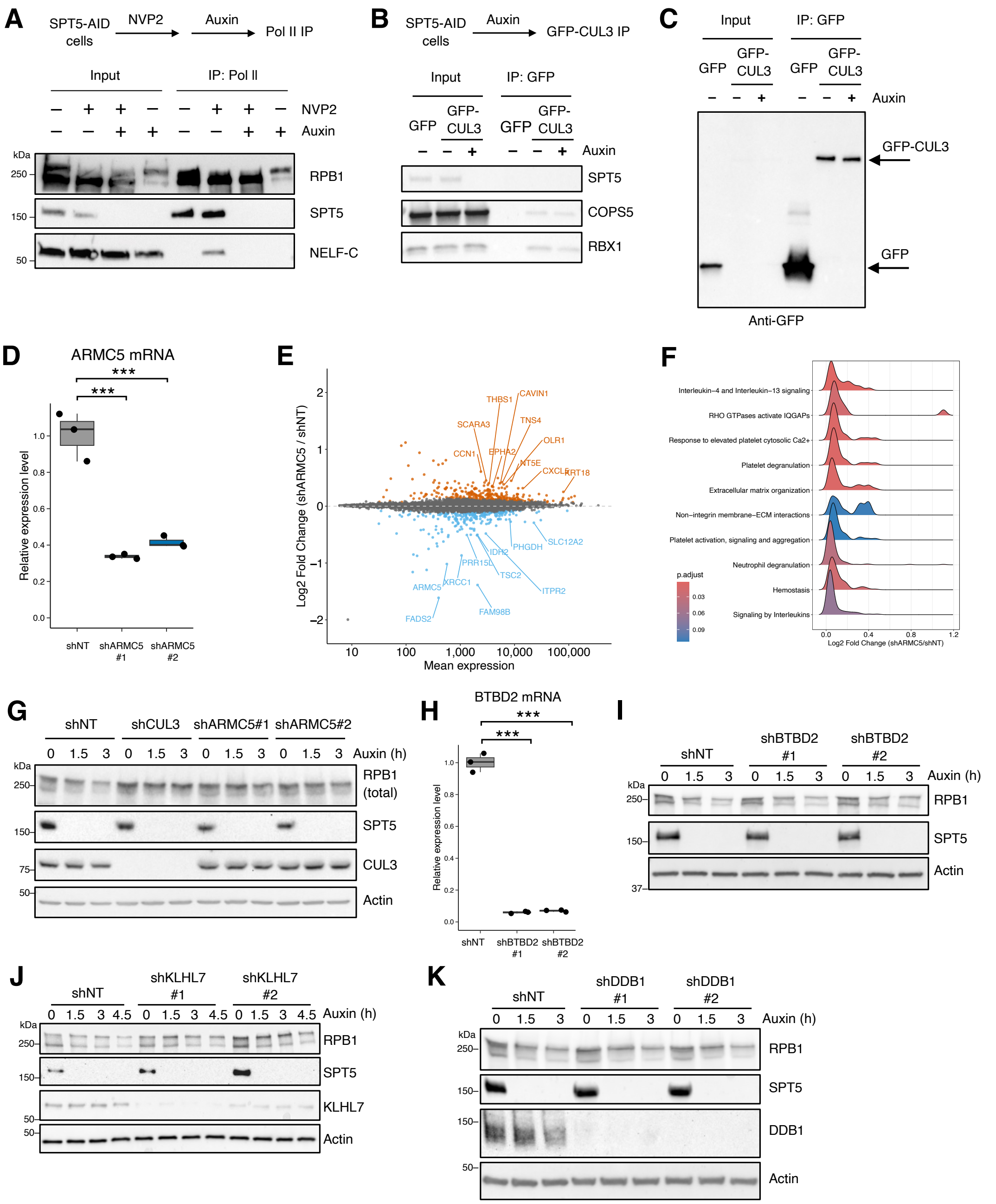

Fig. S1. Analysis of Pol II-associated BTB proteins. (**A**) Western blot analysis of Pol II IP in SPT5-AID cells treated with NVP-2 (250 nM, 2h), followed by auxin (500 μM, 3h). (**B, C**) Western blot analysis of GFP-CUL3 IP in SPT5-AID cells that express GFP-CUL3 constructs, treated with auxin (500 μM, 3h). Expression of the GFP control or GFP-CUL3 was induced by doxycycline. (**D**) RT-qPCR results for ARMC5 knockdown in SPT5-AID cells are shown in box plot. $n = 3$. (**E**) MA plot showing log2 fold change versus mean expression in SPT5-AID cells with ARMC5 shRNA knockdown compared to the non-targeting (NT) shRNA control. $N = 14{,}291$, $n = 3$. Transcripts with adjusted P value < 0.1 are shown in orange or blue. (**F**) Gene set enrichment analysis (GSEA) for the differentially expressed genes in (**D**). The color scale indicates adjusted P values for enrichment. (**G-K**) Analysis of RPB1 stability. In (**G**,**I**,**J**,**K**) Western blot analysis of whole-cell extracts from SPT5-AID cells treated with the indicated shRNA constructs followed by time-course auxin treatment (500 μM). In (**H**), RT-qPCR results are shown for BTBD2 knockdown in SPT5-AID cells. $n = 3$. $^{***}P < 0.001$ (Tukey's test).

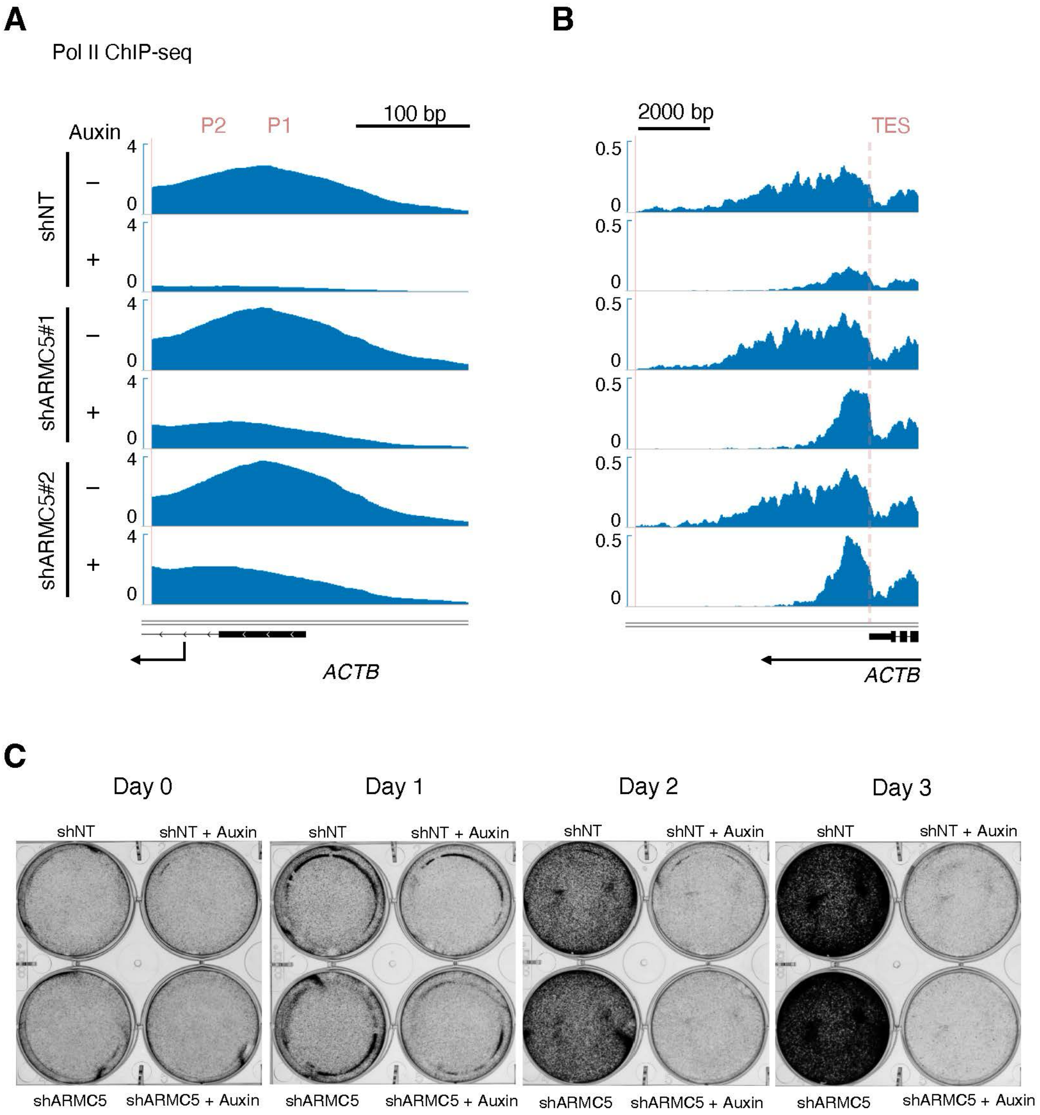

Fig. S2. Analysis of ARMC5 shRNA knockdown in SPT5-AID cells. (**A**, **B**) Close views of representative signal tracks at TSS (**A**) and TES (**B**) for Pol II ChIP-seq in SPT5-AID cells treated with ARMC5 shRNA followed by auxin (500 μM, 3h). P1: the first pause site; P2: the second pause site. (**C**) Crystal violet staining for SPT5-AID cells treated with ARMC5 shRNA followed by auxin (500 μM). Auxin was added to culture on Day 0 and cell growth was monitored every 24h.

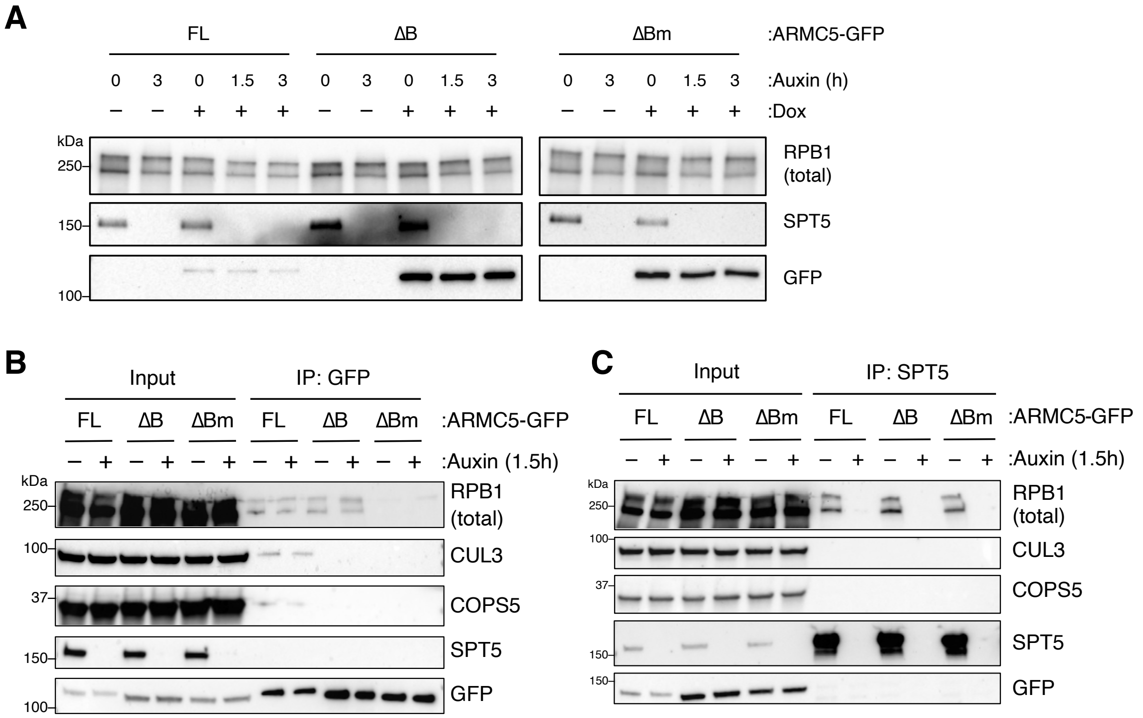

Fig. S3. Analysis of SPT5-AID cells expressing ARMC5-GFP constructs. (**A**) Western blot analysis of whole-cell extracts from SPT5-AID cells with time-course auxin treatment (500 μM). Full-length (FL), BTB domain-deleted (ΔB), or BTB domain-deleted with R593W mutation (ΔBm) ARMC5-GFP construct expression was induced by doxycycline. (**B**) The original blots for Fig. 3C showing the co-IP efficiency. (**C**) SPT5 immunoprecipitation showing no appreciable CUL3-ARMC5 ligase complex. Prior to immunoprecipitation, ARMC5-GFP-expressing SPT5-AID cells were treated with auxin (500 μM, 1.5h).

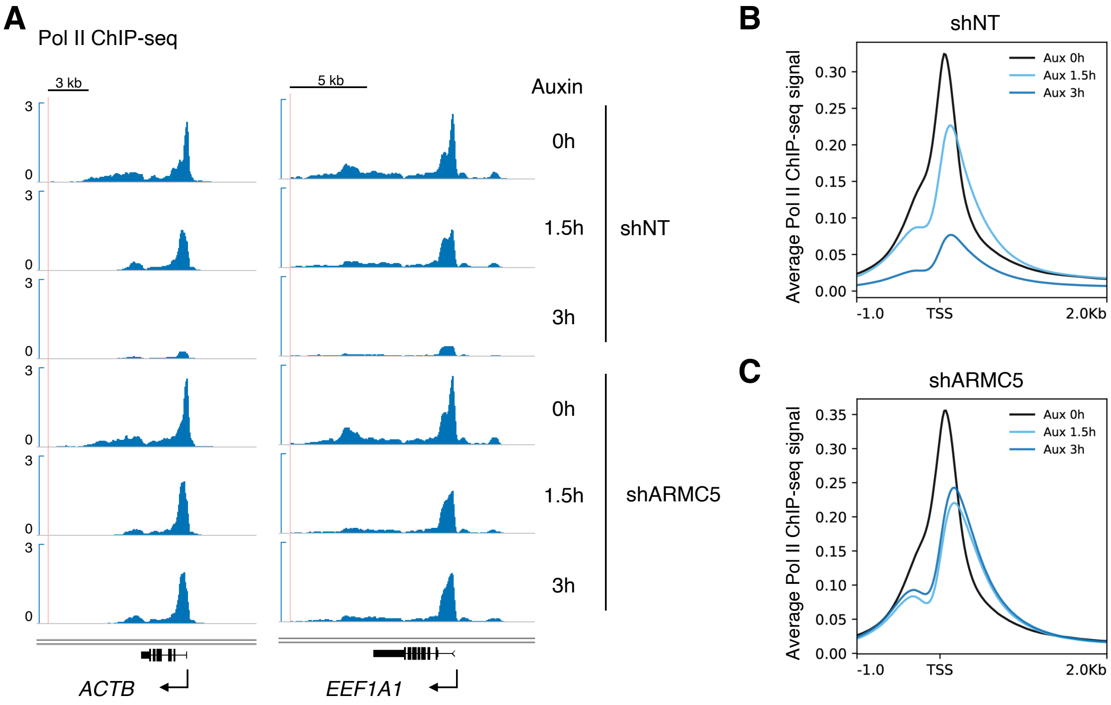

Fig. S4. Analysis of time-course Pol II ChIP-seq data. (**A-C**) Representative signal tracks (**A**) and TSS-centered plots of average signal (**B, C**) for Pol II ChIP-seq in time-course auxin treatment. SPT5-AID cells were treated with ARMC5 shRNA followed by auxin (500 μM) for 0, 1.5h, or 3h.

Table S1. Quantification of Pol II IP and GFP-CUL3 IP MS data. Log-transformed protein abundances in IP-MS data, related to Fig. 1D and 1E, are shown in the spreadsheet (a separate .xlsx file). For Pol II IP-MS, the data was normalized to the Pol II-specific core subunits (see Materials & Methods). For GFP-CUL3 IP, the result of statistical analysis for the +/- auxin conditions is also shown.

Antibody	Application	Source	Identifier
RPB1 NTD [D8L4Y]	WB and ChIP-seq	Cell Signaling Technology	Cat# 14958, RRID: AB_2687876
RPB1 CTD [4H8]	IP	Cell Signaling Technology	Cat# 2629, RRID: AB_2167468
Phospho-RPB1 CTD (Ser2/Ser5) [D1G3K]	WB	Cell Signaling Technology	Cat# 13546S, RRID: AB_2798253
GFP-Trap Magnetic Agarose beads	IP	Proteintech	Cat# gtma, RRID: AB_2631358
SPT5	WB and IP	BD Biosciences	Cat# 611107, RRID: AB_398420
CUL3	WB	Bethyl	Cat# A301-109A, RRID: AB_873023
GFP [B-2]	WB	Santa Cruz	Cat# sc-9996, RRID: AB_627695
GFP [5G4]	ChIP-seq	Cell Signaling Technology	Cat# 55494, RRID: AB_3101977
COPS5 [D15G6]	WB	Cell Signaling Technology	Cat# 9444, RRID: AB_2797702
NELF-C [D5G6W]	WB	Cell Signaling Technology	Cat# 12265, RRID: AB_2797862
β-Actin [8H10D10]	WB	Cell Signaling Technology	Cat# 3700S, RRID: AB_2242334
KHLH7	WB	Invitrogen	Cat# PA5-56167, RRID: AB_2643184
RBX1 [D3J5I]	WB	Cell Signaling Technology	Cat# 11922, RRID: AB_2797769
DDB1	WB	Cell Signaling Technology	Cat# 5428S, RRID: AB_10634753
CDK9 [D-7]	WB	Santa Cruz Biotechnology	Cat# sc-13130, RRID: AB_627245
Spike-in antibody	ChIP-seq	Active Motif	Cat# 61686, AB_2737370
HRP-linked anti-rabbit	WB	Cytiva	Cat# NA934, RRID: AB_772206
HRP-linked anti-mouse	WB	Cytiva	Cat# NA931, RRID: AB_772210

Table S2. Antibodies used in this study.
